# Supplementary material for: Reconciling Mining with the Conservation of Cave Biodiversity: A Quantitative Baseline to Help Establish Conservation Priorities
Source: PLoS One. 2016 Dec 20;11(12):e0168348. doi: 10.1371/journal.pone.0168348 (PMC5173368; doi:10.1371/journal.pone.0168348)
Supplement: S1 Dataset — (ZIP) [file pone.0168348.s002.zip › Taxa/Serra Norte/SN_2013/N5S/Lista N5S-11.pdf]

CAVIDADE N5S-0011

Classe	Ordem	Fam/Outros	Gên/Outros	Espécie	S-U	U-S	Zon.
Annelida	Oligochaeta			jovem	6		P/A
Arachnida	Acari	Bdellidae		sp.1	X		E
Arachnida	Acari	Ixodidae		jovem	X		A
Arachnida	Acari	Labidostommatidae		sp.53	X		A
Arachnida	Acari	Trombidiformes		sp.49	X		P/A
Arachnida	Acari			jovem		X	
Arachnida	Amblypygi	Phrynidae	*Heterophrynus*	*longicornis*	3	X	A
Arachnida	Araneae	Barychelidae		sp.1	2		P
Arachnida	Araneae	Caponiidae		sp.1	X		P
Arachnida	Araneae	Ctenidae	*Ctenus*	sp.1	6		E/P
Arachnida	Araneae	Drymusidae	*Drymusa*	*spelunca*	X		E/P
Arachnida	Araneae	Mysmenidae		jovem	X		E
Arachnida	Araneae	Ochyroceratidae	*Ochyrocera*	sp.1	X	X	P/A
Arachnida	Araneae	Ochyroceratidae	*Speocera*	sp.1	X		P/A
Arachnida	Araneae	Pholcidae	*Mesabolivar*	*eberhard*		X	
Arachnida	Araneae	Pholcidae	*Mesabolivar*	sp.1	X		E/P
Arachnida	Araneae	Pholcidae	Ninetinae	sp.1	X		P
Arachnida	Araneae	Salticidae		sp.7	X	X	E
Arachnida	Araneae	Scytodidae	*Scytodes*	jovem	2		E
Arachnida	Araneae	Theraphosidae	aff. *Holothele*	sp.1	5		E
Arachnida	Araneae	Theridiidae	*Theridion*	sp.1	X		P
Arachnida	Araneae	Theridiidae	*Theridion*	sp.2	X		E
Arachnida	Araneae	Theridiidae	*Theridion*	sp.3	X		E
Arachnida	Araneae	Theridiosomatidae	*Plato*	sp.1	X	X	E/P/A
Arachnida	Araneae	Uloboridae		sp.1	X		E
Arachnida	Araneae	Uloboridae		sp.2	X		E
Arachnida	Opiliones	Escadabiidae		sp.2	X	X	A
Arachnida	Opiliones	Escadabiidae		sp.5	X		P/A
Arachnida	Opiliones	Manaosbiidae	*Saramacia*	sp.1	1		E
Arachnida	Opiliones	Sclerosomatidae	*Prionostema*	sp.1	2		E/P
Arachnida	Opiliones	Stygnidae	*Protimesius*	*gracilis*	3		E/P
Arachnida	Palpigradi	Eukoeneniidae	*Allokoenenia*	sp.1	X		P
Arachnida	Palpigradi	Eukoeneniidae	*Eukoenenia*	sp.1	X		P
Arachnida	Pseudoscorpiones	Chernetidae	*Spelaeochernes*	sp.1	X	X	P
Chilopoda	Scolopendromorpha	Scolopendridae	*Rhoda*	jovem	1		E
Diplopoda	Polydesmida	Chelodesmidae		sp.3	3		P
Diplopoda	Polydesmida	Fuhrmannodesmidae		jovem	X		E/P
Diplopoda	Polydesmida	Pyrgodesmidae		sp.1	X	X	P/A
Entognatha	Collembola	Paronellidae		sp.1	X		P
Entognatha	Collembola	Paronellidae		sp.4	X		P/A
Entognatha	Collembola	Sminthuroidea		sp.2	X	X	A
Entognatha	Diplura	Campodeidae		sp.1	X		E/P
Insecta	Blattodea	Blattellidae		sp.5	7	X	A
Insecta	Coleoptera	Carabidae	*Coarazuphium*	sp.n.2	X		P/A
Insecta	Coleoptera	Chrysomelidae		sp.8	X	X	E
Insecta	Coleoptera	Curculionidae	Scolytinae	sp.2	X		E
Insecta	Coleoptera	Dytiscidae		sp.5	X		A
Insecta	Coleoptera	Dytiscidae		sp.7	X		A
Insecta	Coleoptera	Ptiliidae		jovem	X		E

Insecta	Diptera	Chironomidae		sp.	X		P/A
Insecta	Diptera	Conopidae	*Stylogaster*	sp.	X		A
Insecta	Diptera	Culicidae	*Culex*	sp.	X		P
Insecta	Diptera	Nematocera		sp.		X	
Insecta	Diptera	Phoridae	Metopininae	sp.	X		P
Insecta	Diptera	Psychodidae	*Sciopemyia*	*sordellii*	X	X	
Insecta	Diptera	Sciaridae		sp.	X		A
Insecta	Diptera	Tipulidae	Tipulinae	sp.	X		E
Insecta	Heteroptera	Dipsocoroidea		sp.	X		P
Insecta	Heteroptera	Reduviidae	Emesinae	sp.1	2		P/A
Insecta	Heteroptera	Reduviidae	Emesinae	sp.4	1		P
Insecta	Heteroptera	Reduviidae	Reduviinae	sp.	6		E/P
Insecta	Heteroptera	Veliidae	*Paravelia*	jovem	X	X	P/A
Insecta	Homoptera	Auchenorrhyncha		sp.		X	
Insecta	Homoptera	Cixiidae		jovem	X	X	P
Insecta	Hymenoptera	Formicidae	*Camponotus*	sp.		X	
Insecta	Hymenoptera	Formicidae	*Camponotus*	sp.1	X		P
Insecta	Hymenoptera	Formicidae	*Camponotus*	sp.2	X		E/P
Insecta	Hymenoptera	Formicidae	*Hypoponera*	sp.1	X	X	E
Insecta	Hymenoptera	Formicidae	*Pachycondyla*	sp.2	X		P
Insecta	Hymenoptera	Formicidae	*Paratrechina*	sp.1	X	X	P
Insecta	Isoptera	Termitidae	*Embiratermes*	sp.	X		E/P
Insecta	Isoptera	Termitidae	*Nasutitermes*	sp.	X	X	E/P
Insecta	Lepidoptera	Tineoidea		sp.1	X		A
Insecta	Orthoptera	Gryllidae		sp.	1		P
Insecta	Orthoptera	Phalangopsidae	*Paraclodes*	sp.	10	X	E
Insecta	Orthoptera	Phalangopsidae	*Phalangopsis*	sp.	88	X	A
Insecta	Psocoptera	Epipsocidae	*Epipsocus*	sp.2	X		P
Insecta	Psocoptera	Psyllipsocidae	*Psyllipsocus*	sp.1	X		E
Insecta	Thysanura	Ateluridae		sp.2	X		P
Insecta	Thysanura	Nicoletiidae		jovem	X		E/P
Gastropoda	Pulmonata	Subulinidae	*Leptinaria*	sp.		X	
Gastropoda	Pulmonata	Systrophiidae	*Happia*	sp.		X	
Malacostraca	Isopoda	Armadillidae		sp.1	X		P
Malacostraca	Isopoda	Philosciidae		sp.2	X		E
Malacostraca	Isopoda	Scleropactidae	*Circoniscus*	*buckupi*	X		A
Amphibia	Anura	Bufonidae	*Rhinella*	sp.	2		P
Amphibia	Anura	Leptodactylidae	*Leptodactylus*	cf. *vastus*		X	
Mammalia	Chiroptera	Emballonuridae	*Peropteryx*	*kappleri*	15		P/A
Mammalia	Chiroptera	Furipteridae	*Furipterus*	*horrens*	5		P/A
Mammalia	Chiroptera	Phyllostomidae	*Carollia*	*perspicillata*	1		P
